# Effect of Multiple Micronutrient Fortification on Physico-Chemical and Sensory Properties of Chhash (Traditional Indian Yogurt-Based Drink)

**DOI:** 10.3390/foods8010005

**Published:** 2018-12-22

**Authors:** Shashank Gaur, Anna W. Waller, Juan E. Andrade

**Affiliations:** 1 Food Science and Human Nutrition, University of Illinois, Urbana-Champaign; Urbana, IL 61801, USA; gaur2@illinois.edu (S.G.); awaller2@illinois.edu (A.W.W.); 2 Division of Nutritional Sciences, University of Illinois, Urbana-Champaign; Urbana, IL 61801, USA

**Keywords:** fortification, malnutrition, micronutrient, fermented product

## Abstract

Introduction: Micronutrient malnutrition is a persistent problem in India mainly due to low dietary diversity and poor nutrient density of staple foods. The addition of a commercial micronutrient mix in terms of physico-chemical and sensory characteristics was evaluated. Methods: The yogurt prepared with reduce-fat milk (2%), 20 g/L CHN-11 fermentation culture and 12 h incubation (42 °C) was diluted at a rate of 35% to yield a 32 mg/100 mL concentration. The premix provided vitamin A, vitamin D, folic acid, iodine, iron, and zinc oxide to cover ~20–35% recommended RDA (recommended dietary allowance) per serving (250 mL). A three-factorial design, 2 treatments (control and fortified), 3 temperatures (4, 25, and 40 °C) and 4 time points (1, 2, 3 and 6 days), was used to analyze the physico-chemical properties in terms of titratable acidity, pH, color, and viscosity. A discriminatory sensory test (triangle) was performed with college students (*n* = 58) and mothers with young children (*n* = 25), who were living in Mehsana, India to evaluate the difference between fortified and non-fortified cooled (4 °C) product. Results: Fortification did not affect most of the physico-chemical properties of chhash. As expected, titratable acidity increased whereas pH decreased with increasing storage time and temperature. Fortified samples showed higher b* values, whereas L* and a* were not affected. Viscosity changed due to temperature and time, but not fortification. Participants could not discriminate between samples in terms of color, aroma, and taste. Conclusion: Fortification of chhash is technically feasible.

## 1. Introduction

Micronutrient malnutrition or the deficiency of essential vitamins and minerals afflicts close to 1.5 billion people worldwide, especially in India where a large proportion of the population lives in poverty and food insecurity [1]. Micronutrient deficiencies are associated with unacceptably high morbidity and mortality in children and pregnant women. More than 70% of Indian population consumes less than 50% recommended dietary allowance (RDA) of micronutrients, typically due to inadequate food intake, poor nutrient bioavailability (inhibitors, preparation method, interactions), and/or the presence of infections [2]. Vitamin A deficiency affects 57% of children under five and their mothers and results in the death of 333,000 children every year. Anemia results in a yearly loss of lives of 22,000 pregnant women. Every year, around 200,000 infants are born with neural tube defects as a result of folic acid deficiency [3]. Micronutrient malnutrition results in poor physical and mental development in children, losses in productivity among adults, and an increased vulnerability to infectious diseases [4].

The fortification of foods, especially of dairy products, is a well-established and successful practice in many high- and low-income countries, which contributes to effectively overcoming micronutrient deficiencies [5,6]. Although India is the largest producer of milk in the world, fortified dairy products are limited in Indian marketplaces. Nevertheless, in response to a newly implemented “Food Fortification Regulation,” dairy products such as Vitamins A and D fortified milk are now emerging in the Indian markets. The Food Safety and Standards Authority of India (FSSAI) currently recommends fortification of milk with 270–450 µg of Vitamin A (retinyl acetate or palmitate) and 5.0–7.5 µg of Vitamin D (cholecalciferol and ergocalciferol from plant sources) per liter of milk to provide 15–30% of the daily requirements [7]. 

Dairy products such as flavored milk, yogurt, and ice cream are examples of market-driven fortification vehicles, however, there is limited evidence on the fortification of other traditional products. Chhash is a traditional, low-cost, fermented dairy product in India. Chhash, also known as sour cultured buttermilk, contains 1% fat, 5.5% solids not fat (SNF) and minimum acidity of 0.8% lactic acid (equivalents). Chhash is widely consumed in India for its palatability, thirst-quenching characteristic and its potential therapeutic value based on its probiotic content [8,9]. The shelf life of a pasteurized chhash packaged in plastic pouches (polyethylene) is 48 h when stored below 8 °C. 

Fortification of dairy products with micronutrients is known to impact the physico-chemical and sensory properties of dairy products [10,11]. The objective of this study was to systematically evaluate the effect of chhash fortification on the physical-chemical and sensory characteristics stored at different temperature conditions.

## 2. Materials and Methods 

### 2.1. Materials

Organic Horizon homogenized reduced-fat milk (2%) was purchased at a local supermarket, Champaign, Illinois. Yogurt culture CHN-11 mesophilic aromatic was donated by CHR Hansen (Milwaukee, WI, USA). Fortitech yogurt micronutrient premix (Table 1) was donated by DSM, Gurgaon, India. Sodium hydroxide and Phenolphthalein indicator were procured from Sigma-Aldrich (St. Louis, MO, USA). Mason jars of 250 mL were procured from Ball (Broomfield, CO, USA).

### 2.2. Design

A three factorial—2 treatments (control and fortified), 3 temperatures (4, 23 and 40 °C) and 4 time points (1, 2, 3 and 6 days) was utilized to analyze the effect of fortification on the physico-chemical properties of chhash.

### 2.3. Culture Taxonomy

The culture taxonomy of CHN-11 culture was *Lactococcus lactis* subsp. *cremoris*, *Leuconostoc*, *Lactococcus lactis* subsp. *lactis*, *Lactococcus lactis* subsp. *lactis* biovar *diacetylactis*. This culture was selected to match the culture generally used by the Indian dairy companies (Dudhsagar Dairy, Mehsana, GJ, India) for preparation of chhash.

### 2.4. Chhash Preparation 

Chhash was prepared using a method adapted from Dudhsagar Dairy (Mehsana, GJ, India). Milk (1.5 kg, 2% fat, and 9% SNF) was heated on the stovetop to 42 °C, measured using a handheld thermometer. Culture (30 g, CHN-11) was added to the milk to achieve 20 g culture per liter, and stirred continuously for 5 ± 0.5 min until the culture was thoroughly incorporated into the milk. Next, 100 mL of the milk/culture mix was distributed into 12 mason jars (250 mL each) and was immediately capped and placed in an incubator preheated to 42 °C for 12 h to set the yogurt and achieve a titratable acidity between 0.3 and 0.5% and a pH < 5. After incubation, the yogurt was equally distributed into two 1000 mL beakers to have 600 mL of yogurt in each beaker. The beakers were labeled “control” (unfortified) and “fortified”. To prepare chhash, the yogurt was diluted with double deionized water (DDI) by adding 210 mL DDI to 600 mL yogurt and thoroughly mixed for 5 min using a handheld mixer. To prepare fortified chhash at 32 mg fortificant per 250 mL, 104 mg of premix was thoroughly mixed (2 min) with a 100 mL aliquot of chhash from the “fortified” beaker followed by mixing it back into the large beaker (810 mL) and sonicating in a water bath for 2 minutes. Finally, control and fortified chhash (135 mL) was transferred to six mason jars and capped. Two jars from each treatment were placed in the refrigerator (4 ± 0.5 °C), two were placed in a dark box (to prevent vitamin degradation due to light exposure) at room temperature (23 ± 2 °C), and two were placed in an incubator (40 ± 2 °C) for each time point. The protocol was repeated to prepare samples for each time point (day 1, 2, 3 and 6).

### 2.5. pH and Titratable Acidity 

The pH and titratable acidity were measured using Method number 981.12 and Method 947.05 of the Association of Analytical Communities (AOAC), respectively [12]. Briefly, the pH meter was calibrated, the probe was placed in the middle of the sample and the reading was recorded after reaching stabilization. The titratable acidity was analyzed by filling a 100 mL burette with 0.1 N NaOH. Then, 10 g of chhash, 30 mL deionized distilled water, and 1 mL phenolphthalein were mixed in a 250 mL Erlenmeyer flask. The mixture was swirled underneath a constant stream of NaOH until a pink endpoint was held for at least 10 seconds. Titratable acidity (expressed as % lactic acid) was calculated using Equation (1),

(1) $$\mathrm{Titratable}\;\mathrm{acidity}\;(\mathrm{as}\;\%\;\mathrm{lactic}\;\mathrm{acid})=\;\frac{\;V\;\times \;N\;\times \;W\;\times \;100}{\mathrm{MW}\;\times \;1000}$$

where V is the volume in mL of 0.1N NaOH used in titration; N is the normality of NaOH used in titration; MW is the molecular weight of lactic acid (90 g/mol); and W is the weight of the sample.

### 2.6. Color

The color of the chhash samples was measured using the HunterLab (Labscan XE, Hunter Associates Laboratory, Inc., Reston, VA, USA). Briefly, all samples were brought to room temperature (25 ± 2 °C) before testing. Chhash sample (15 mL) was poured into a Petri dish (100 mm × 15 mm) and measured in terms of L* (lightness) and a*b* (chroma). Calibration using standard reference plates prior to sample analysis was conducted. The absolute measure of change in color was calculated as the ΔE-76 value using Equation (2),

(2) $$∆E*\;=\sqrt{{{(L}_{f*}{\;-\;L}_{c*})}^{2}{\;+\;(A}_{f*}{\;-\;A}_{c*}{)}^{2}{\;+\;(B}_{f*}{-\;B}_{c*}{)}^{2}}$$

where, L_f*_, A_f*_ and B_f*_ are the L*, a*, and b* values of fortified samples, and L_c*_, A_c*_ and B_c*_ are L*, a*, and b* values of the control sample. The equation integrates the values for L*, a* and b* into a single formula to determine total perceivable color difference based on reference [13] as shown in Table 2.

### 2.7. Viscosity 

The viscosity of chhash samples was measured using a Brookfield viscometer (Model DV-II Pro, Brookfield Engineering Laboratories, Middleboro, MA, USA) with an RV2 spindle. A chhash sample (130 mL) at a temperature 5 ± 1 °C was placed in 250 mL beaker and was analyzed at 60 rpm after 30 s of shear [14]. The samples were analyzed on day 0 and day 6.

### 2.8. Sensory Test 

Research protocols used in this study were similar to those recently reported by Gaur et al. 2018 and were approved by the Institutional Review Board at the University of Illinois, Urbana-Champaign [15]. The study was conducted in collaboration with the Mansinhbhai Institute of Dairy and Food Technology (MIDFT), Mehsana, India. Untrained individuals, 58 college students (18–24 years) and 25 mothers (18–45 years) with young children, all of whom were otherwise healthy (no diarrhea, cold, or fever), were randomly recruited from MIDFT and within a 20 km radius from MIDFT, respectively. The number of assessors was calculated using a sensitivity test at α-risk (strength of evidence that the difference was apparent) of 0.05, β-risk (strength of evidence that the difference was not apparent) of 0.05 and *p*_d_ (maximum allowable proportion of distinguishers) of 50% to keep the amount of tasting within reasonable limits. Written consents were obtained from the participants on the day of the tests. Mothers with young children were enrolled in this study as women and children (>5 years of age) population are most vulnerable to micronutrient deficiencies and Indian mothers normally seek and provide food and care for young children, who are also at homes. Students from MIDFT are considered a convenient sample as the intention of the test was to identify by sensory evaluation the presence of micronutrients in the fortified chhash samples. Thus, the purpose of this test was to assess whether subjects could detect any difference between the control and fortified chhash using a triangle sensory test [16]. Chhash samples were prepared one day in advance in a food grade lab at MIDFT and were transferred to the test site under refrigerated conditions (4 ± 2 °C). Panelists were served one tray at a time with three samples of chhash samples (2 ± 0.5 g) placed in random order and identified only by three-digit codes. One of the samples contained fortified chhash and the other two samples contained the control or vice versa. When presented with the samples, the panelists were requested to look at the samples and select the odd sample. Next, the tray was replaced with another set of three coded samples and the panelists were requested to smell and select the odd one. Finally, the tray was replaced one more time and the panelists were asked to rinse their palate with water (25 ± 2 °C) and taste the sample and select the odd one. The samples were maintained at 4 ± 2 °C and all tests were performed in well-lit and ventilated rooms. All testing sessions were scheduled between 11:00 AM and 1:00 PM. The subjects who were able to identify the different sample (correct) were given a score of 1 whereas the others (incorrect) were given a score of 0. The total number of correct responses was then compared with the critical number at α = 0.05, calculated using equation (3),

(3) $$Z_{\mathrm{critical}}=\frac{k-\frac{1}{3}\times n}{\sqrt{\frac{2}{9}\times n}}$$

where k is the minimum number of correct responses to reject the assumption of “no difference”, *n* is the total number of participants [16].

### 2.9. Statistics 

Statistical analyses were performed using SAS (SAS Institute, Cary, NC, USA). All experiments were conducted in duplicates, and the results are reported as mean ± SD. For all analyses, determinations were made in triplicate as independent experiments. Data were analyzed by a three-way analysis of variance (ANOVA) (treatment × storage temperature × storage time) for titratable acidity and pH outcomes, two-way ANOVA (treatment × storage temperature) for viscosity outcomes. The lack of fit test *F*-values were used to reflect if the models were significant and coefficient of variance less than 10% was used to establish that the experiments were conducted with reasonable accuracy and suggesting that the models can be reproducible. The Tukey’s Honest Significant Difference test was used to compare means. Statistical significance was established at an alpha of 0.05. 

## 3. Results

### 3.1. pH and Titratable Acidity

**Figure 1:**
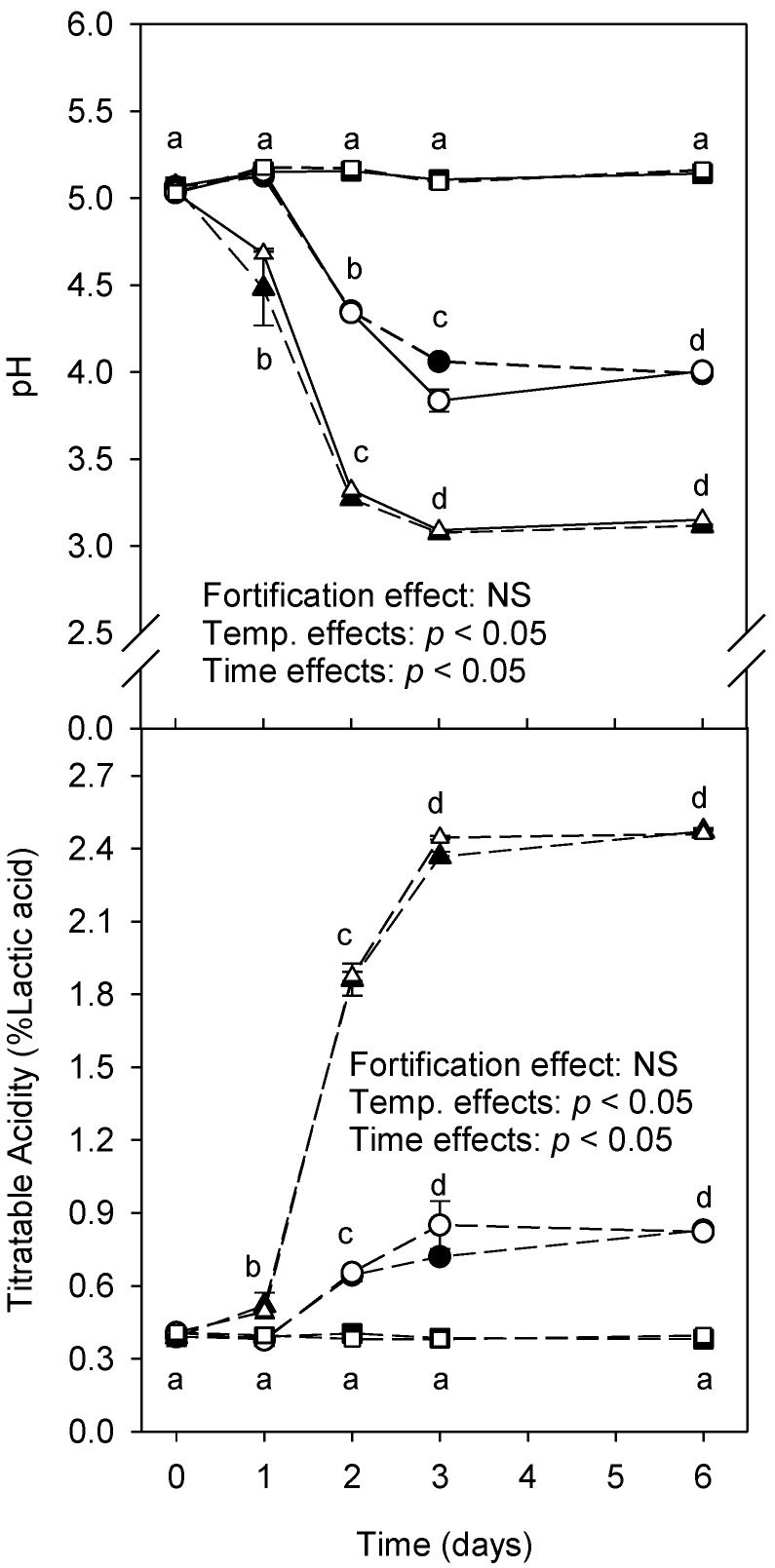
Changes in pH and titratable acidity of control and fortified chhash samples over 6 days stored under refrigeration (squares; 4 ± 1 °C), room temperature (circles; 23 ± 2 °C), and in an incubator (triangles; 40 ± 2 °C). Data points represent means ± SD. Different superscripts (a, b, c, d) indicate significant differences across temperature x time interactions (repeated measures ANOVA; *p* < 0.05).

Micronutrient fortification had no effect on the titratable acidity and pH at any given temperature and time, except for day 3 where the titratable acidity of control and fortified samples was slightly different (*p* > 0.05; Figure 1, bottom graph). Both storage temperature and time point (day) and their respective interactions were significant (*p* < 0.05) for both titratable acidity and pH. The titratable acidity for both fortified and control samples did not change over time for samples stored at 4 °C. Control and fortified samples showed high titratable acidity (*p* < 0.05) after several days of storage at 23 and 40 °C. Similarly, the pH of samples did not change after storage at 4 °C. Samples showed a reduced pH after several days of storage at 23 and 40 °C (Figure 1, top graph).

### 3.2. Color

**Figure 2:**
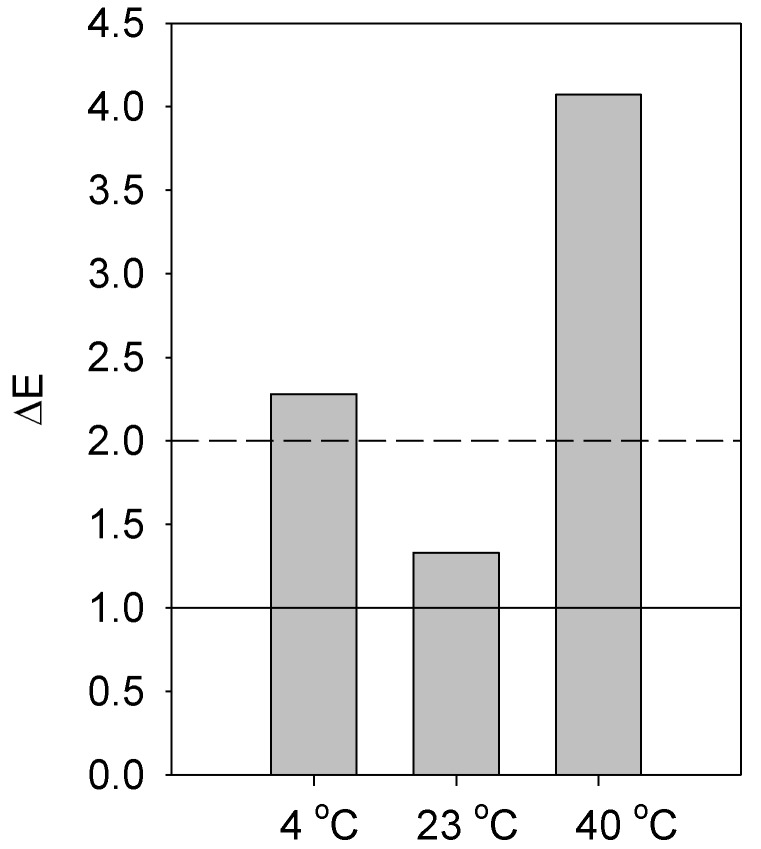
Perception of color difference between fortified and control samples (ΔE) stored under refrigeration (4 ± 1 °C), at room temperature (23 ± 2 °C), or in an incubator (40 ± 2 °C). The dashed and solid lines indicate perceptible at a glance (above dashed line) and not perceptible by human eyes (below the solid line). Between the dashed and solid lines indicate perceptible through close observation (See Table 2).

Fortified samples had higher b values for color (*p* < 0.05) compared to the control samples across all time points and temperatures, suggesting an increased yellowness due to fortification. L* and a* color values were not different across treatments, times, and temperatures. Based on Table 2 (i.e., ΔE-76), fortified samples stored at 4 and 40 °C can be perceived at a glance by the human eye (Figure 2). 

### 3.3. Viscosity

**Figure 3:**
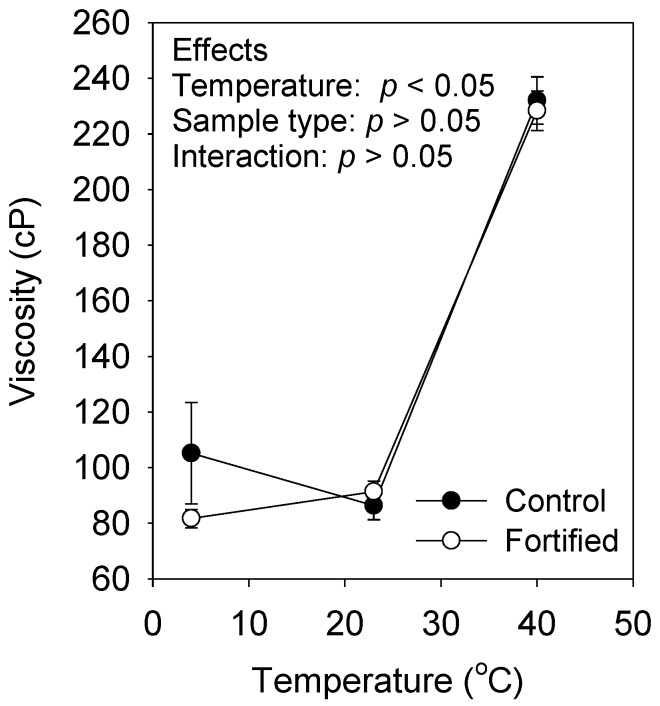
Apparent viscosity (Brookfield viscometer) of control and fortified chhash samples measured after six days of storage under refrigeration (4 ± 1 °C), at room temperature (23 ± 2 °C), an in an incubator (40 ± 2 °C). Data points represent means ± SD. *p*-values shown correspond to statistical analyses using two-way ANOVA for treatment and temperature factors, and their interactions.

Micronutrient fortification had no effect on the viscosity of samples stored at all temperatures on day 6 (Figure 3). After 6 days of storage at 40 °C, samples showed increased viscosity (*p* < 0.05, range 235.3 to 228.3 cP). Viscosity did not change among samples stored at 4 and 23 °C (*p* > 0.05, range 81.6 to 105.1 cP). 

### 3.4. Sensory Test

**Figure 4:**
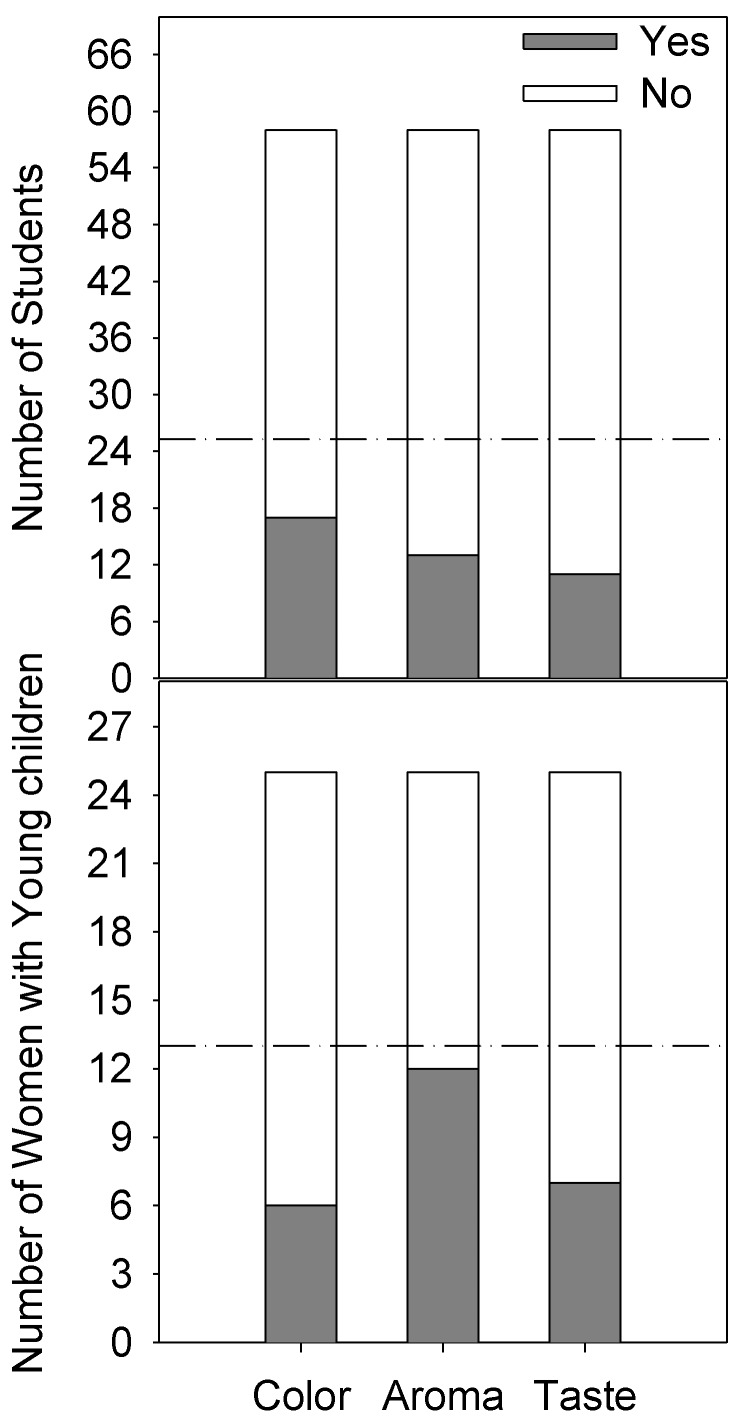
Sensory evaluation showing no differences (*p* < 0.05; sensitivity levels: *p*_d_ = 50%, α = 0.05, β = 0.05) between control and fortified samples in terms of color, aroma, and taste as evaluated by students (*n* = 58) and mothers with young children (*n* = 25) in Mehsana, India.

Figure 4 shows that both student and mothers with young children were not able to discriminate between the control and fortified chhash samples, given the sensitivity levels selected for the test (*p*_d_ = 50%, α = 0.05, β = 0.05).

## 4. Discussion

Fortification is a nutrition specific strategy which has been implemented worldwide to address micronutrient deficiencies [17]. Extensive evidence supports the effectiveness of the use of staples such as salt, cereal flours, milk, and sugar for the successful delivery of iodine, iron, folic acid, and vitamin A to a diverse population [18,19,20,21]. Despite its long-standing application, more than 2 billion people continue to suffer from hidden hunger [22]. One of the many factors that hinder fortification success is the technical feasibility of the fortificant and the chosen product, which encompasses the consumption pattern, the level of fortification, the stability of premixes, the interactions among nutrients, and the physical-chemical changes that could affect final product’s acceptability and use [23]. In this study, the authors sought to address some of these limitations by proposing the fortification of chhash, a traditional fermented dairy product widely consumed in India. This study was performed in the light of a newly passed Food Safety and Standards Regulations (2017) [7] on the fortification of dairy products with the specific objective of exploring the effect of a micronutrient premix on the physico-chemical properties of chhash.

The manufacture of dairy fermented products includes a variety processing and ingredient technologies, which results in their distinctive textures, colors, mouthfeels, and sour flavors [24]. Despite its importance, the addition of micronutrients into foods cannot succeed at the expense of modifications in flavor, to standards of identity (e.g., pH, TA), or any other characteristics that make products like chhash a unique drink. The addition of the micronutrient premix to chhash did not result in changes in its physical-chemical characteristics, even at temperatures not recommended for storage. Previous, related studies on the fortification of dairy products showed no effect on titratable acidity and pH [25,26,27,28]. Fortification of yogurt with Vitamin A and C did not change pH, titratable acidity, or sensory characteristics of yogurt samples stored at 3 °C for 6 weeks [25]. The addition of micro- or nanosized iron, zinc, and calcium into yogurt did not affect pH or titratable acidity after 28 days at 4 °C [28]. Vitamin D has been reported to be stable in yogurt during processing and storage [29]. In agreement with those studies, the present results indicate that micronutrient fortification had no significant effect on titratable acidity and pH at any given temperature. As expected, storage temperature and time increased titratable acidity and reduced pH. This has been shown by other groups, in which the exposure of yogurt to higher temperature over several hours or days allows for further microbial activity beyond the initial sample preparation time of 12 h [30,31,32]. Previous studies report similar findings of yogurt increasing titratable acidity and decreasing pH with storage at room temperature or above. Because of these changes in product quality, it is important for the consumer to properly refrigerates these products. Similarly, there was no change in apparent viscosity after micronutrient fortification. However, the samples stored at 40 °C increased their apparent viscosity. This is due to increased microbial activity, which continued the fermentation process and resulted in protein denaturation and gelation [33].

Chhash presents a white, milky color. Addition of micronutrients can unduly influence food’s color. The addition of the micronutrient premix influenced the color toward light yellow. One study found that fortification of chocolate milk with sodium ferric pyrophosphate did not change color as determined with a color difference meter initially or after 14 days holding at 4 °C [10]. This may be due, however, to the natural brown color of chocolate milk, which could obscure a change in yellowness. Although the fortification caused a significant change in chhash yellowness, the sensory study results indicate that a change in color is not perceivable to the target consumer population. Moreover, in several regions of India, fermented products are mixed with spices [9], which could potentially mask any sensory modification due to micronutrient fortification. 

The present findings indicating no sensory difference between fortified and unfortified chhash are in agreement with a previous study, in which both a trained and a consumer panel detected no significant difference in terms of appearance, mouthfeel, flavor, or overall quality on a nine-point hedonic scale when evaluating iron fortified yogurt [34]. In another study, chocolate milk fortified with sodium ferric pyrophosphate produced little or no off-flavors initially or after holding at 4 °C for 14 days [10]. As the present work was conducted using a mixed consumer panel (i.e., mothers with young children and young adults of both sexes), it can be speculated that other consumers would accept both products equally, however, this requires further evaluation.

A limitation of this study is that the contents of the micronutrients added were not measured after exposure to time and temperature conditions. Nonetheless, prior evidence demonstrates and recommends the storage of fermented products under refrigeration to maintain their nutritional content (mainly of vitamins) and physico-chemical attributes before their consumption. In those studies, some micronutrients (i.e., vitamins C, A and D) losses occurred due to exposure to broad wavelength light, temperature and time [25,35,36].

## 5. Conclusions

The addition of micronutrients to chhash would bear no technical and sensory barriers to its current format of consumption. Furthermore, the results of this study indicate that this fortified dairy product would be feasible and acceptable to enter the market under the current Food Safety and Standards Regulations (Fortification of Foods). Studies evaluating processing feasibility of the fortification of chhash at a pilot or large-scale are needed.

## Figures and Tables

**Table 1:** Composition of yogurt premix (per 32 mg in 250 mL of product) and proportion of recommended dietary allowance.

Nutrient	Unit	Amount	RDA ^1^	% RDA Covered ^2^
Vitamin A (Retinol Acetate)	µg	150	400|700	37.5|21.4
Vitamin D2 (ergocalciferol)	µg	3	15|15	20|20
Folic Acid	µg	27.8	200|400	14.0|7.0
Iron (Ferric Pyrophosphate)	mg	4.2	10|8	42.0|52.5
Zinc (Zinc oxide)	mg	2.33	5|11	46.6|21.2
Iodine (Potassium Iodide)	µg	22.5	90|150	25|15

^1^ From the USA Food and Nutrition Board, RDA for children (4–8 years)|men (19–70 years); ^2^ Calculated as the amount of nutrient from 1 portion size (250 mL) divided by RDA for each age group; RAD, recommended dietary allowance.

**Table 2:** Correlation between ΔE-76 values and human eye perception.

ΔE*	Human Eye Perception
≤1.0	Not perceptible by human eyes
1–2	Perceptible through close observation
2–10	Perceptible at a glance
11–49	Colors are more similar than opposite
100	Colors are the exact opposite
